# 

**Published:** 1897-07

**Authors:** 


### CONTENTS.

ORIGINAL COMMUNICATIONS. PAGE
Reminiscences of Sixty-four Years of Practice. By OTIS AVERY, D.D.S., Honesdale, Pa. . . . . 421
A Chapter in Dental History and Bibliography. By WILLIAM H. TRUEMAN, D.D.S., Philadelphia . . . . . 431
Thoughts on Regulating. By HOWARD E. ROBERTS, D.D.S., Philadelphia . . . 435
Defective Articulation accompanied by Pain corrected by opening the Bite and the Insertion of Immovable Bridges. By J. G. BRIGIOTTI, Paris, France . . 439
Some Dental Manifestations of Gout. By J. S. GILLIAMS, M.D., D.D.S., Philadelphia . . . . . 441
ABSTRACTS AND TRANSLATIONS.
Formaldehyde . . . . . 444
REPORTS OF SOCIETY MEETINGS.
American Academy of Dental Science . . . . . 449
The New York Institute of Stomatology . . . . . 457
EDITORIAL.
American Dental Association and Reorganization . . . . . 467
The American Medical Association . . . . . 471
Editors not always Responsible . . . . . 472
Eighty-nine and in Practice . . . . . 474
Old Point Comfort . . . . . 475
The Death of Dr. Emile Magitot . . . . . 475
OBITUARY.
Le Docteur Magitot . . . . . 476
Resolutions of Respect.—Dr. Frank Abbott . . . . . 479
Professor Dr. Med. Ludw. Hollænder . . . . . 480
DOMESTIC CORRESPONDENCE.
An Incident in Dr. Garretson's Life . . . . . 480
Reply to Dr. C. N. Pierce . . . . . 481
Correction . . . . . 482
CURRENT NEWS.
American Dental Association . . . . . 482
National Association of Dental Faculties . . . . . 482
National Association of Dental Examiners . . . . . 483
New Jersey State Dental Society . . . . . 483
New England Association of Dental Examiners . . . . . 484

